# Cholec80-CVS: An open dataset with an evaluation of Strasberg’s critical view of safety for AI

**DOI:** 10.1038/s41597-023-02073-7

**Published:** 2023-04-08

**Authors:** Manuel Sebastián Ríos, María Alejandra Molina-Rodriguez, Daniella Londoño, Camilo Andrés Guillén, Sebastián Sierra, Felipe Zapata, Luis Felipe Giraldo

**Affiliations:** 1 Department of Electric and Electronic Engineering, Universidad de Los Andes, Bogotá D.C., Colombia; 2 Proelium SAS, Bogotá D.C., Colombia; 3 Department of General Surgery, Universidad CES, Medellín, Colombia; 4 Department of Biomedical Engineering, Universidad de Los Andes, Bogotá D.C., Colombia

**Keywords:** Cholecystitis, Cholelithiasis

## Abstract

Strasberg’s criteria to detect a critical view of safety is a widely known strategy to reduce bile duct injuries during laparoscopic cholecystectomy. In spite of its popularity and efficiency, recent studies have shown that human miss-identification errors have led to important bile duct injuries occurrence rates. Developing tools based on artificial intelligence that facilitate the identification of a critical view of safety in cholecystectomy surgeries can potentially minimize the risk of such injuries. With this goal in mind, we present *Cholec80-CVS*, the first open dataset with video annotations of Strasberg’s Critical View of Safety (CVS) criteria. Our dataset contains CVS criteria annotations provided by skilled surgeons for all videos in the well-known Cholec80 open video dataset. We consider that Cholec80-CVS is the first step towards the creation of intelligent systems that can assist humans during laparoscopic cholecystectomy.

## Background & Summary

Laparoscopic cholecystectomy is one of the most common surgeries performed in the world. For example, in the United States, around 20 million people suffer from gallbladder diseases that imply nearly 300,000 cholecystectomy surgeries per year. Despite being considered a low-risk surgery, bile duct injuries (BDIs) have occurred at a constant rate in the last 30 years^1^, leaving devastating consequences on the affected patients. Strasberg and colleagues^2^ showed that most BDIs occur due to miss-identification of the bile duct and the cystic duct. This fact encouraged Strasberg to propose a method known as Critical View of Safety (CVS), which provides criteria to correctly identify cystic structures before the complete removal of the gallbladder. However, even though this identification method is commonly used and accepted for surgeons as an important attempt to reduce bile duct miss-identifications^3^, Way *et al*. showed that around 97% of the BDI occurrences were due to visual perceptual illusions and, in most of the cases of BDIs, surgeons did not even know what the problem was^4,5^. These findings encourage the development of technologies that aid surgeons to accurately identify the cystic structures during surgery and therefore to reduce BDI occurrence rates.

This type of technologies for automatic detection and identification are not new in the context of laparoscopic surgeries. For example, researchers have studied the problems of surgical instrument segmentation and tracking^6–8^, surgery phase identification^9^ and anatomical structure localization^10^. Most of the current work leverage complex pre-trained convolutional neural network architectures and handcrafted databases annotated by experts in the medical field^11^. In particular, the automatic detection of CVS for BDI prevention has gained recent interest from several researchers. The work of Mascagni and colleagues^12^ aimed to detect critical views of safety on still images carefully annotated and selected by skilled surgeons. They used binary annotations of the occurrence of CVS criteria and anatomical segmentation annotations, and trained a deep neural network using both types of annotations to predict CVS occurrence. Tokuyasu *et al*.^13^ tackled the problem in an object detection setting by detecting four important anatomical landmarks: the common bile duct, the cystic duct, the lower edge of the left medial liver segment, and the Rouviere’s sulcus. The correct identification of these four landmarks has proven to help surgeons to avoid bile duct injuries. In this case, the authors used the fully-convolutional architecture YOLO and bounding box-like annotations. On the other hand, Madani *et al*.^14^ addressed the problem of identifying safe and dangerous zones of dissection as well as anatomical landmarks during laparoscopic cholecystectomy as a segmentation problem providing real-time intra-operative guidance.

Despite the above mentioned studies and the effort of Mascagni and colleagues in proposing a reproducible method for objective video reporting of CVS in laparoscopic videos^15^, there are not publicly available datasets with annotations of the CVS criteria that can be used by researchers interested in addressing the problem of automatic CVS identification. Annotating videos based on CVS criteria on videos of laparoscopic cholecysectomy procedures is a challenging task that is time consuming and must be done only by skilled surgeons. In this data descriptor, we introduce *Cholec80-CVS*, the first open dataset with annotations based on CVS criteria on all the videos in the well-known Cholec80 video dataset^11^. Cholec80 is a popular open dataset that contains 80 video recordings of cholecystectomies performed by 13 surgeons and that has been widely used to study artificial intelligence-based models for real time analysis in surgeries.

## Methods

### Strasberg’s criteria in the critical view of safety

Strasberg originally proposed three main criteria over a given still image that allow the surgeon to determine if it is safe to proceed with the clipping and cutting process for gallbladder removal, and thus avoid bile duct injuries^16^. Strasberg’s method, known as Critical View of Safety, is based on three criteria: first, only two structures can be clearly seen connected to the gallbladder; second, the lower one third of the gallbladder is separated from the liver to expose the cystic plate; third, the hepatocystic triangle must be completely clear of tissue allowing proper visibility of all cystic structures.

### Cholec80 dataset

Cholec80 dataset contains 80 high-quality videos of gallbladder laparoscopic surgeries. It was constructed by researchers from the research group CAMMA at University of Strasbourg, France. Videos in Cholec80 were recorded at 25 frames per second, and all videos were recorded for different patients, surgeons, and light conditions, facilitating the application of learning methods for surgery analysis. Additionally, each video was labeled frame by frame according to the surgical phase and the surgical instruments in the scene. Cholec80 has been widely used in recent research^8,17–19^, establishing it as one of the most popular benchmarks for research in laparoscopic cholecystectomy.

### Cholec80-CVS: annotations of CVS criteria

Our annotators strictly followed a scoring system to ensure the creation of a high quality dataset. This system was originally proposed by Sanford’s and Strasberg’s^16^ and latter used by Mascagni and colleagues^15^ to annotate Strasberg’s CVS on still images. However, we extended it by adding a new set of complementary rules to reduce the uncertainty during the annotation process.

For each video in Cholec80, we analyzed the *preparation* and *Calot’s triangle dissection* phases. Our annotators carefully selected video segments where at least one of the non-zero Strasberg criteria was satisfied. Then, a score of one or two was assigned to the video segment following the scoring system proposed by Sanford^16^ and a complementary set of rules for each of the criteria proposed by our annotators as described in Tables 1–3. The annotators only considered video segments where the presence of the criteria was consistent for at least three seconds, allowing for the presence noise induced by occlusions or abrupt camera movements during this time lapse. We consider that all frames contained in the annotated video segment share the same annotation. Therefore, it is possible to get annotations for each frame. Those frames with a score of 0 are denoted as negative examples, while the other ones are considered as positive examples. We compute the total score of an image by adding up the individual scores of each criterion. A view of safety is achieved when the total score is equal to or greater than 5.

**Table 1:** Set of rules used to produce consistent and reliable annotations for the two structures criteria.

**Two structures criteria**	
**2 Points**	
	- Two structures can immediately and clearly be seen connecting to the gallbladder.
	- The two structures are clearly visible despite partial occlusions caused by the surgical instruments. *
	- It is clear that the structures are present but can not be seen entirely due to the camera’s zoom.*
**1 Point**	
	- Two structures can be seen connecting to the gallbladder, but there is some overlap of duct and artery or a technical feature, such as poor lighting or lack of color contrast, that interferes with clarity of determination. Photograph requires study to make assessment.
	- The structures are clear but the camera lenses are dirty or the image is slightly blurred. *
	- There is a lot of adipose tissue or the structures need more dissection. *
**0 Points**	
	- Due to overlap or technical issues 2 separated cystic structures cannot be seen.
	- There are more or less than two structures. *
	- The artery was cut and only the cystic duct is visible. *

**Fig. 1:**
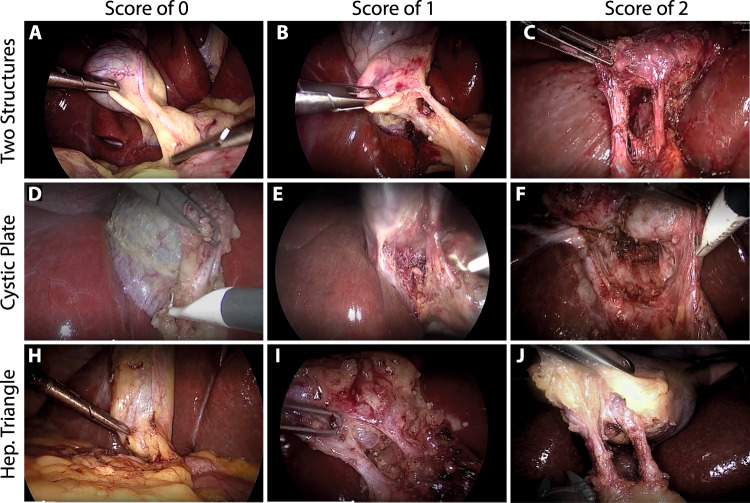
Reference images for the Strasberg criteria.

Superscript * indicates that the rule was proposed by our annotators, and the other ones are the original rules proposed by Sanford and Strasberg^16^. Reference images are provided in Fig. 1a–c.

**Table 2:** Set of rules used to produce consistent and reliable annotations for the cystic plate criteria.

**Cystic plate criteria**	
**2 Points**	
	- Cystic plate is immediately clearly visible to approximately its bottom one third.
	- Excellent dissection and traction that allows for clearly seeing the cystic plate. *
	- The liver can be seen throughout the dissection. *
**1 Point**	
	- Cystic plate is visible but overlapped by other structures so that it is not optimally seen or an insufficient amount of the plate is shown. Photograph requires study to make assessment.
	- It is clear that the cystic plate is visible and there is no tissue left to be dissected. However, the camera axis must be calibrated. *
**0 Points**	
	- Cystic plate not visible due to positioning, light, obstruction of view by instruments, or coverage with clot.
	- The cystic plate needs more dissection. *

Superscript * indicates that the rule was proposed by our annotators, and the other ones are the original rules proposed by Sanford and Strasberg^16^. Reference images are provided in Fig. 1d–f.

**Table 3:** Set of rules used to produce consistent and reliable annotations for the Hepatocystic triangle criteria.

**Hepatocystic triangle criteria**	
**2 Points**	
	- Hepatocystic triangle is cleared of tissue so that visibility of cystic structures and plate are completely unimpeded, but also so that viewer can be immediately certain than no other structures are in the triangle.
	- It is possible to see two structures, a part of the gallbladder and a perfectly dissected cystic plate. *
	- There is no redundant tissue in the Calot’s triangle and the liver can be seen throughout the dissection. *
**1 Point**	
	- Somewhat less than the whole triangle can be clearly seen or technical issues reduce ability to see optimally. Photograph requires study to make assessment.
	- The cystic plate or the structures criteria have a score of 1. *
	- The calot’s triangle or the cystic plate need more dissection.*
	- The camera axis needs to be calibrated.*
**0 Points**	
	- Tissue in triangle obscures view of cystic structures cystic plate and does not allow conclusion that that there are no other structures in triangle. Or technical issues prevent determination of how well cleared the triangle is.
	- More dissection is needed in both the calot’s triangle and in the cystic plate. *

Superscript * indicates that the rule was proposed by our annotators, and the other ones are the original rules proposed by Sanford and Strasberg^16^. Reference images are provided in Fig. 1h–j.

We are aware that annotating time-lapses may induce noise, given that low quality frames can occur within an annotated video segment. For instance, brief occlusions and blurred or over exposed images may occur due to the abrupt camera movements that are common in laparoscopic cholecystectomy. However, we consider that Cholec80-CSV is the first step towards the creation of intelligent systems that can assist humans during surgeries. Therefore, it is crucial to model noisy input data to build robust systems in real scenarios.

## Data Records

Cholec80 Videos can be obtained from the University of Strasbourg’s research group CAMMA official web site http://camma.u-strasbg.fr/datasets. On the other hand, our annotations are publicly available as a XLSX file^20^. Each row in this file details the initial and final frame of a video segment where our annotators detected any of the Strasberg’s criteria as well as the score assigned to it. All video segments that are not in the provided dataset are considered to have a 0 score for all criteria. Table 4 describes each data column in our database.

**Table 4:** Description of each data column present on Cholec80-CVS database.

Column	Description
Video	Video Number [1–80]
Critical View	Binary flag that indicates the presence of a critical view
Initial Minute	Initial minute where the event of interest occurred
Initial Second	Initial second where the event of interest occurred
Final Minute	Final minute where the event of interest occurred
Final Second	Final second where the event of interest occurred
Two Structures	Score of the two structures criteria.
Cystic Plate	Score of the cystic plate criteria.
Hepatocystic Triangle	Score of the hepatocystic triangle criteria.
Total	Total score of the video segment

## Technical Validation

### Experience of the annotators

Our annotators were highly skilled surgeons: one of them with a record of approximately 700 successful cholecystectomies performed, while the second has performed about 470 successful cholecystectomies. Each of the surgeons was paired with one surgical resident. Even though residents had prior experience in laparoscopic cholecystectomy, they also received additional training from the experienced surgeons. It consisted of weekly meetings before starting the annotation process. During these meetings, the annotators carefully studied the original set of rules proposed by Sanford and Strasberg to detect CVS. Additionally, the annotators proposed a complementary set of rules shown in Tables 1–3 seeking consistency in the annotations. Each team evaluated 40 videos independently. When one of the teams considered their annotation unreliable, the final score was defined after a discussion between the four annotators.

### Analysis of the annotations

**Fig. 2:**
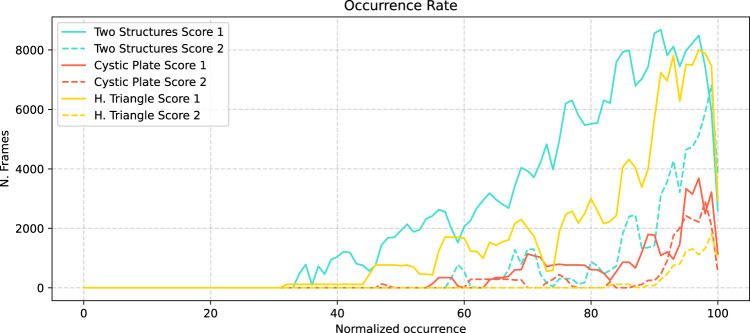
Normalized temporal occurrence of each criterion in all videos. The horizontal axis corresponds to a normalized time line, where 0 is a the beginning of the surgery and 100 is the instant when clipping and cutting is performed.

In contrast to the methodology proposed by Mascagni^15^ where only the last 60 seconds before clipping and cutting phase were annotated, we analyzed the entire portion of the video prior the clipping and cutting phase. Figure 2 shows the relative occurrence of the Strasberg’s criteria across all 80 videos. The horizontal axis corresponds to a normalized time line, where 0 is the beginning of the surgery and 100 is the instant when clipping and cutting is performed. The vertical axis indicates the total number of frames across all 80 videos that contain each Strasberg’s criterion as explained in the methods section. Note that all criteria are heavily concentrated in the last quarter of the process, particularly for a score of two. However, a considerable large amount of frames annotated with a score of one occur in the second half of the analyzed phases. The latter suggest that analyzing a small video segment of 60 seconds before clipping and cutting can potentially lead to a considerable loss of important data samples.

It is evident the high imbalance between scores in the dataset. Given that most of the time a view of safety is achieved just for a couple of seconds before the gallbladder dissection, frames tagged with scores of two are very scarce. This behavior is consistent with the dynamics of a typical laparoscopic cholecystectomy and the methodology followed in the work of Mascagni and colleagues^15^.

**Fig. 3:**
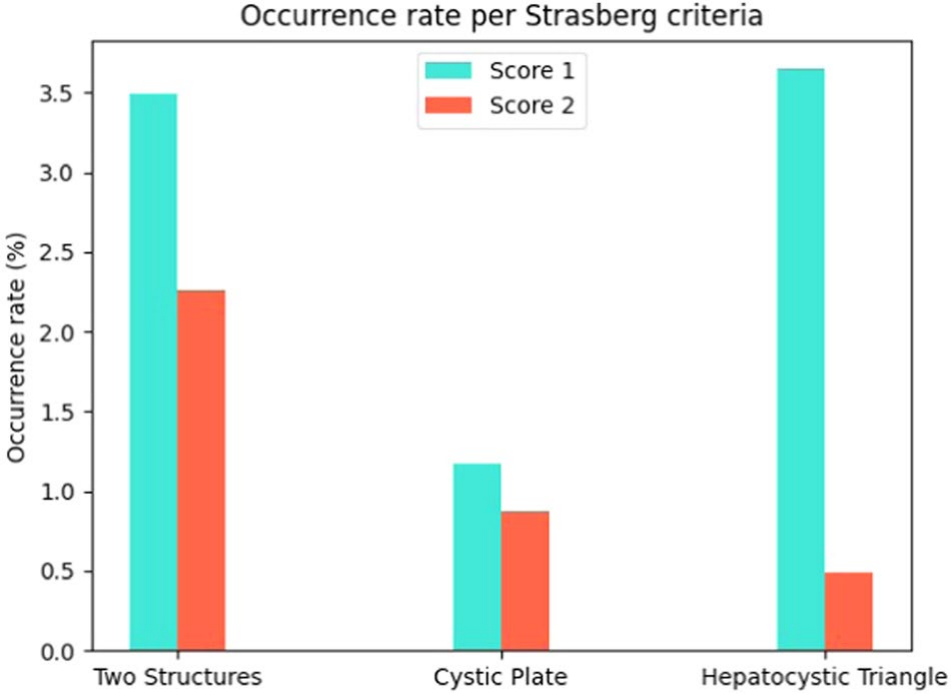
Strasberg Criteria’s occurrence rate in the *preparation* and *Calot’s triangle dissection* phases. These results show a considerable data imbalance between different criteria.

Figure 3 shows the low occurrence rates of these frames for each of the Strasberg criteria, indicating a strong data imbalance between the frames annotated with score of zero and all the other frames. It is also evident an imbalance between classes, being the cystic plate criteria a clear example of an under represented class. Given that Cholec80-CVS provides annotations of the starting and ending seconds where non-zero scores are present instead of frame-wise annotations, abrupt camera movements or partial camera occlusion may occur briefly during the annotated time frame, inducing noise to the database since they can be annotated as positive samples.

## Usage Notes

To ease the use of Cholec80-CVS we provide public access to both the database and all the code related to this work. The database is publicly available in XLSX format^20^. Cholec80-CVS can be used for researchers to assess novel image classification architectures to automatically detect CVS criteria. We also encourage researchers to contribute to enriching the current dataset either by refining the current version or by adding new samples.

### Limitations

Cholec80-CVS dataset has some important limitations. First, abrupt camera movements are very common, creating blurry low quality frames. Some of them may occur within the time-lapse annotated by the surgeons, producing noisy samples. Additionally, due to the nature of the surgery, in some frames a criteria may be briefly occluded by actions performed by the surgeon, but the frame will still be considered as a positive sample since it occurs within the annotated time-lapse. This behavior suggests possible research directions, for instance, implementing video analysis techniques. Analyzing multiple subsequent frames may reduce the negative impact of these noisy samples as well as reduce the probability of miss-classify images due to temporal occlusions.

The second limitation of Cholec80-CVS is the high data imbalance due to the nature of the surgery. As it is shown in Fig. 3, there is an significant imbalance between different criteria. Moreover, there is also an important imbalance between the annotations of the same criterion, as in the case of the hepatocystic triangle. In general, positive samples have very low occurrence rates, and even in some Cholec80 videos the CVS is never achieved.

## Data Availability

We provide scripts to transform our annotations to the frame-wise labels and also the source code of some baseline models that use standard deep learning techniques to detect CVS criteria using our database for interested users. All these scripts were coded using Python 3.8.11 and Pytorch as the machine learning framework. All scripts were tested on Linux Machines. The repository README file contains detailed instructions to ease the use of the repository and brief descriptions of all files. The code is publicly available at https://github.com/ManuelRios18/CHOLEC80-CVS-PUBLIC, licensed under MIT OpenSource license. Therefore, permission is granted free of charge to copy and use this software and its associated files.
